# Simultaneous Enantioseparation of Three Chiral Antifungal Pesticides by Hydroxypropyl-*γ*-CD-Modified Micellar Electrokinetic Chromatography

**DOI:** 10.1155/2023/9993526

**Published:** 2023-10-09

**Authors:** Xiaoyu Yang

**Affiliations:** Qingdao Huanghai University, Qingdao 266427, China

## Abstract

Simultaneous enantioseparation of three commonly used chiral antifungal pesticides (diniconazole, hexaconazole, and imazalil) was first studied based on micellar electrokinetic chromatography (MEKC) with hydroxypropyl-*γ*-CD (HP-*γ*-CD) as chiral selector. In this study, the importance of experimental parameters such as chiral selector type and concentration, sodium dodecyl sulfate (SDS) concentration, the ratio of methanol, and separation voltage in optimizing were investigated. The simultaneous enantioseparation of diniconazole, hexaconazole, and imazalil was successfully achieved in 30 mM borate buffer (pH 9.0) containing 10 mM HP-*γ*-CD and 20 mM SDS with methanol (8%) added as organic modifiers. The resolution of diniconazole, hexaconazole, and imazalil was 15.2, 2.12, and 2.78, respectively, and the peak efficiency (N) was over 566,825 plates/m. This study provides an alternative way to systematically separate chiral antifungal pesticides with high efficiency.

## 1. Introduction

Nowadays, developing green pesticides [1] has become a hotspot in the field of chemistry and environmental research since traditional pesticides have heavily affected environmental safety. Chiral pesticide [2] is one of the most important green pesticides because of its high efficacy, small dosage, and safety for the environment. While the *R*-enantiomer of chiral pesticides showed great difference compared to *S*-enantiomers in biological activity, environmental behavior, and biological toxicity [3], for example, the efficiency of Salithion's *S*-enantiomer was higher than that of *R*-enantiomer for mosquito, armyworm, and mouse; however, the result was contrary to that of housefly [4]. When exposed to 100 and 200 *μ*gL^−1^ of hexaconazole, the (−)-enantiomer was found to accumulate more readily in zebrafish than the (+)-enantiomer, suggesting that hexaconazole has significant enantioselective bioaccumulation [5]. Therefore, the preparation of enantiomeric purity compounds is essential. However, most chiral pesticides were used in their racemic form because of the restriction of technology and cost, which resulted in low efficacy. The key factor in developing chiral pesticides is to establish an effective way to separate and analyze chiral compounds.

Numerous chromatographic methods have been used for enantiomeric separations of pesticides including high-performance liquid chromatography (HPLC) [6, 7], gas chromatography (GC), and capillary electrophoresis (CE) [8]. The chiral column is the fatal factor in separating enantiomers for HPLC and GC, but it is expensive and difficult to separate different kinds of chiral compounds simultaneously. In contrast, MEKC, a new powerful way for enantiomeric separation, has been broadly recognized due to its advantages including simple operation, quick speed, high efficacy, and low consumption, especially for the usage of pseudostationary phases that provide more selectivity for chiral separation compared to traditional CE [9, 10].

**Figure 1:**
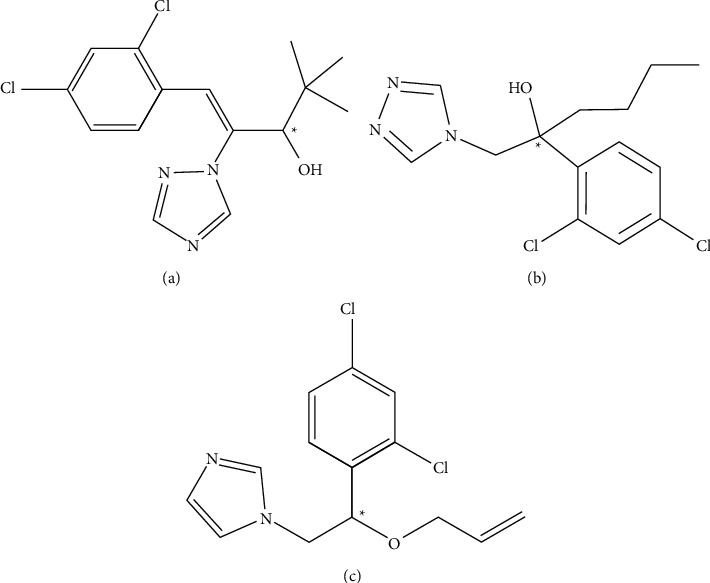
Chemical structures of three chiral antifungal compounds: (a) diniconazole, (b) hexaconazole, and (c) imazalil.

Different kinds of chiral pesticides have been produced, including triazole fungicides, pyrethroid insecticides, and phenoxy herbicides. Diniconazole, hexaconazole, and imazalil are common fungicides used for crop sterilization (Figure 1). Many ways have been performed previously to separate them, but most methods using HPLC or GC could only separate one kind of fungicide with chiral columns [11, 12]. For example, Li et al. [13] successfully resolved imazalil with cellulose tris(3-chloro-4-methylphenylcarbamate) (CCMPC) as a chiral stationary phase, and Chai et al. [14] used cellulose-tris(4-methylbenzoate) (CTMB) as a chiral stationary phase to obtain better resolution. Electrophoresis methods also have been reported to separate chiral substances, for example, the separation of diniconazole by MEKC with both HP-*γ*-CD and hydroxypropyl-*β*-cyclodextrin (HP-*β*-CD) [15, 16] and enantiomeric separation of diniconazole and hexaconazole using sulfated-*β*-CD (S-*β*-CD). Besides, hexaconazole was separated using supercritical fluid chromatography (SFC) [17]. However, although the chiral stationary phase method of high-performance liquid chromatography has high sensitivity, it also has the defects of complex operation and high cost. The methods using SFC are costly and difficult to operate, and electrophoresis methods suffer from poor peak and small resolution. Therefore, it is of practical significance to develop a rapid, simple, high peak value, and high-resolution method for the chiral resolution of chiral antifungal pesticides. To our knowledge, the method using CD-MEKC for the simultaneous separation of diniconazole, hexaconazole, and imazalil has never been reported yet.

In this study, we aimed to establish a simultaneous enantioseparation method of diniconazole, hexaconazole, and imazalil (Figure 1) by using CD-MEKC for the first time. The method could be a technical protocol for the separation of enantiomeric purity compounds. Furthermore, it could reduce the pollution of the environment and harm human beings due to the use of pesticides.

## 2. Materials and Methods

### 2.1. Chemicals and Reagents

Diniconazole, hexaconazole, and imazalil were obtained from Aladdin (Shanghai, China); *β*-CD, HP-*β*-CD, and 2-HP-*γ*-CD were purchased from Aladdin; C_12_H_25_SO_4_Na (SDS), Na_2_HPO_4_, Na_2_B_4_O_7_.10H_2_O, NaOH, H_3_PO_4_, and methanol were obtained from Sinopharm Chemical Reagent (Shanghai, China). All experimental water was purified by a Milli-Q apparatus (Millipore, Bedford, MA, USA). All samples and background solutions were filtered by a 0.22 *μ*m nylon filter (MSI, Westboro, MA, USA) and degassed by sonication.

Background electrolyte was prepared by dissolving Na_2_B_4_O_7_.10H_2_O in ultrapure water to 100 mM. The running buffer was prepared by dissolving CD and SDS in the background buffer and adjusting the pH of butter with NaOH or H_3_PO_4_. All buffers were filtered through a 0.22 *μ*m filter before use. Stocking solutions of signal chiral antifungal pesticides were prepared in methanol at a concentration of 2000 *μ*g/mL and stored at 4°C. Working solutions were prepared by diluting the stocking solutions with a background buffer to a suitable concentration. All samples were filtered before sampling.

### 2.2. Instrumentation

Capillary electrophoresis (CE) with an ultraviolet detector (UV) (Unimicro Technologies, Pleasanton, CA, USA), chromatography workstation (TriSep-2003 chromatography, Unimicro Technologies, Pleasanton, CA, USA), a fused-silica capillary column, ultrasonic cleaner, electronic scales, pH meter, 0.22 *μ*m microporous membrane, and 2 mL sterile syringe were used.

Preconditioning capillary: The capillary was flushed with 0.1 mol/L NaOH for 5 min followed by flushing with ultrapure water for 5 min and running buffer for 10 min before use. The capillary was flushed with 0.1 mol/L NaOH, ultrapure water, and running buffer for 3 min between injections to confirm the stability of the system and the reproducibility of results. At the end of the experiment, the capillary was rinsed for 3 min with ultrapure water to protect the column and UV detector.

The optimal analysis conditions were as follows: capillary: 50 *μ*m × 80 cm, 60 cm effective length; gravity injection 10 s at a height difference of 10 cm; voltage: +13 kV; buffer: 30 mM sodium borate, 20 mM SDS, 10 mM HP-*γ*-CD, and 8% methanol; wavelength: 220 nm; the analysis was performed at room temperature; sample concentrations: 0.1 mg/mL diniconazole, 0.05 mg/mL hexaconazole, and 0.05 mg/mL imazalil.

## 3. Results and Discussion

### 3.1. Selection of Cyclodextrins

CD, as an additive, is commonly used for chiral separation, especially for *α*-CD, *β*-CD, and *γ*-CD. To expand the application range of CD, chemical modifications were introduced by using the derivatization of CD to enhance the enantioselectivity. Many modified CDs have been performed previously, for instance, hydroxypropyl-CD, hydroxymethyl-CD, and sulfobutyl-CD.

The separation abilities of *β*-CD, HP-*β*-CD, and HP-*γ*-CD were studied in the 15 mM sodium borate containing 20 mM SDS with pH from 2 to 9, and all concentrations of CDs were 20 mM. Results showed that neither diniconazole nor hexaconazole could be enantiomerically separated from *β*-CD and HP-*β*-CD. In addition, *β*-CD was barely dissolved in phosphate buffer and turned into crystals after a night. The baseline was abnormal with high noise when HP-*β*-CD was used. We found that all samples were separated effectively by using HP-*γ*-CD, with constant baseline and low noise. Therefore, HP-*γ*-CD was used as a chiral additive for the following experiments.

### 3.2. The Effect of Cyclodextrin Concentration

**Figure 2:**
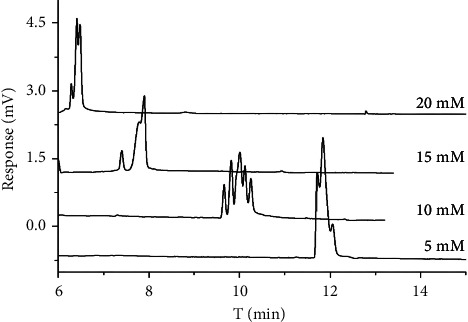
Chromatogram of three chiral antifungal pesticide enantiomers with different HP-*γ*-CD concentrations.

The concentration is an important parameter to control in enantiomeric separation. Migration times and resolution would be changed by the variation in the CD concentration, which impacts the enantiomeric separation. The chiral separation of all chiral antifungal pesticides was studied at 5, 10, 15, and 20 mM HP-*γ*-CD in the 15 mM sodium borate containing 20 mM SDS at pH 9.  Figure 2 shows that the chiral resolution was increased with the HP-*γ*-CD concentration in the range from 5 mM to 10 mM and the chiral resolution was best at 10 mM HP-*γ*-CD, while the chiral resolution of samples was decreased with the HP-*γ*-CD concentration since the concentration was higher than 10 mM. The possible reason is that HP-*γ*-CD has no electric charges, so the moving speed is equal to *μ*_eo_. The content of the sample in CD was increased with the increase of HP-*γ*-CD concentration; accordingly, the content of the sample in SDS was decreased. As a result, the sample's *μ*_app_ was increased, so the retention time of the sample was shorter than before. The inclusion complexes formed by cyclodextrins and chiral substances were partitioned between the pseudostationary phase and aqueous solution. Eventually, the good enantioresolution of the three chiral antifungal pesticides was achieved at 10 mM HP-*γ*-CD, so 10 mM was the optimal concentration for HP-*γ*-CD.

### 3.3. The Effect of SDS Concentration

**Figure 3:**
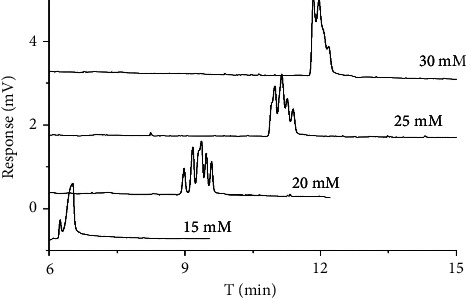
Chromatogram of three chiral antifungal pesticide enantiomers in different SDS concentrations.

The concentration of SDS was also an important parameter in chiral separation. In this study, the effect of varying SDS concentration from 15 to 30 mM in the 15 mM sodium borate containing 10 mM HP-*γ*-CD at pH 9 was also performed to obtain better enantiomeric separation of all chiral antifungal pesticides (Figure 3). It can be seen from Figure 3 that the analysis time of diniconazole, hexaconazole, and imazalil increased from 6 to 12 min when the SDS concentration increased from 15 to 30 mM. The reason was that SDS, as a pseudostationary phase, was negatively charged which led to its migratory direction contrary to electroosmotic flow (EOF). Therefore, the sample was partitioned between SDS micelles and aqueous solution, and the moving speed of SDS was enhanced with the increase of negative charge, while the *μ*_app_ of the sample was reduced when the concentration of SDS was increased. As a result, the retention time of the chiral antifungal fungicide became longer. There was no chiral resolution for all of them at 15 mM SDS, and partial separation of the three enantiomers appeared when the concentration of SDS increased to 20 mM. However, the chiral resolution decreased when the concentration of SDS decreased from 20 to 30 mM. Therefore, the optimal concentration of SDS was 20 mM.

### 3.4. The Effect of Methanol Concentration

**Figure 4:**
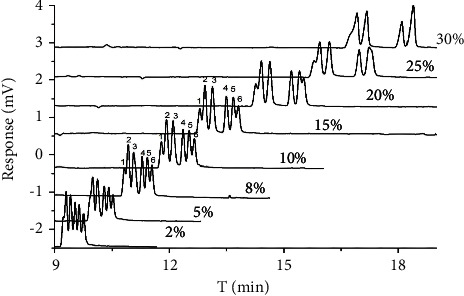
Chromatogram of three chiral antifungal pesticides enantiomers in different methanol volume percentages: (1) diniconazole I, (2) imazalil I, (3) imazalil II, (4) hexaconazole I, (5) hexaconazole II, and (6) diniconazole II.

To achieve high enantioresolution, the ratio of methanol was also investigated. The chiral separation of all chiral antifungal pesticides was studied in the 15 mM sodium borate containing 10 mM HP-*γ*-CD, and 20 mM SDS at pH 9 when the ratio of methanol was 1%, 5%, 8%, 10%, 15%, 20%, 25%, and 30%. As shown in Figure 4, the migration time of the three chiral enantiomers increased with the increase in the methanol ratio. The reason was that the organic solvent reduced the EOF and the sample's *μ*_app_, which led to an increase in analysis time. The chiral resolution of six peaks was changed regularly along with the amount of methanol, and the Rs of imazalil II and hexaconazole I were increased with an increase in the methanol ratio, while the Rs of hexaconazole II and diniconazole II were decreased. The Rs of diniconazole I and imazalil I were good when the ratio of methanol was 8%, 10%, and 15%, and 10% of methanol was the best choice for the following experiment based on the comprehensive consideration of the migration time and chiral resolution.

### 3.5. The Effect of Borate Concentration

**Figure 5:**
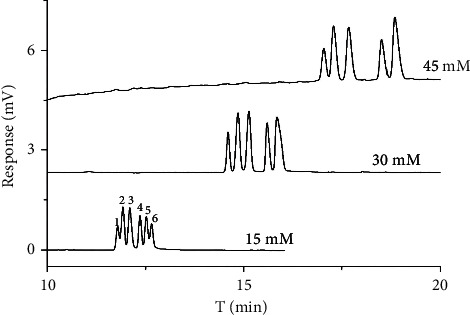
Chromatogram of three chiral antifungal pesticide enantiomers in different sodium borate concentrations: (1) diniconazole I, (2) imazalil I, (3) imazalil II, (4) hexaconazole I, (5) hexaconazole II, and (6) diniconazole II.

The concentration of sodium borate also had a great influence on the chiral resolution; therefore, the enantioresolution of the three chiral antifungal pesticides was studied at 15, 30, and 45 mM sodium borate in sodium borate containing 10 mM HP-*γ*-CD and 20 mM SDS with pH 9 (Figure 5). Baseline separation was successfully achieved for all samples except for hexaconazole II and diniconazole II at 30 mM sodium borate. The current intensity was so high that it may cause joule heat when the concentration of sodium borate increased to 45 mM. In comprehensive consideration, the optimal concentration of sodium borate was 30 mM.

### 3.6. The Effect of Voltage

**Figure 6:**
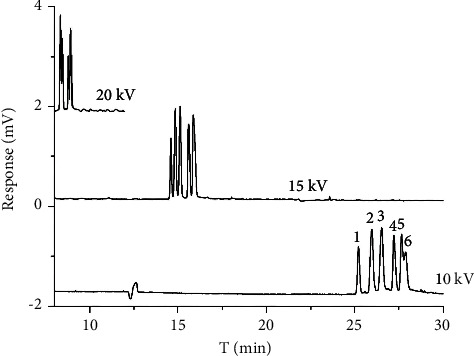
Chromatogram of three chiral antifungal pesticide enantiomers in different voltages: (1) diniconazole I, (2) imazalil I, (3) imazalil II, (4) hexaconazole I, (5) hexaconazole II, and (6) diniconazole II.

The effect of different voltages on the chiral separation was also studied because the separation voltage was the main factor of EOF. The voltage was optimized from 10 to 20 kV. It can be learned from Figure 6 that the separation condition was improved with the reduction of voltage due to the decrease of EOF. 15 kV was chosen preliminarily for chiral separation based on comprehensive consideration of resolution and migration time.

### 3.7. Optimization Considerations for MEKC Chiral Separation Conditions

**Figure 7:**
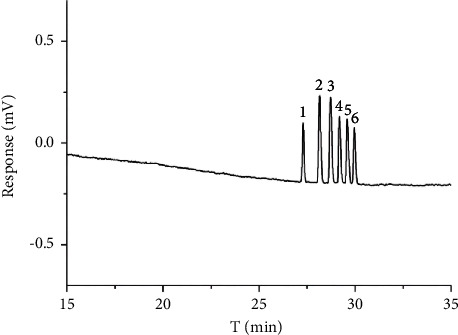
Chromatogram of three chiral antifungal pesticide enantiomers in optimum condition: (1) diniconazole I, (2) imazalil I, (3) imazalil II, (4) hexaconazole I, (5) hexaconazole II, and (6) diniconazole II.

Not all enantiomers had been successfully separated yet after a series of optimization experiments, and the separation of hexaconazole II and diniconazole II remained the main problem. From the results of methanol optimization, the separation resolution increased with the reduction of methanol, so the ratio of methanol was reduced from 10% to 8%. Meanwhile, considering that the reduction of EOF might improve the resolution and a low capillary inside diameter (ID) could improve the peak profile and reduce the EOF and Joule heat, the capillary column was changed from 75 *μ*m ID with an effective length of 40 cm (80 cm total length) to 50 *μ*m ID with an effective length of 60 cm (80 cm total length), and the separation voltage was decreased to 13 kV. The three pairs of chiral antifungal pesticides were ideally separated, and the resolution between two adjacent peaks was all more than 1.80. The chromatogram in optimal conditions can be seen in Figure 7.

### 3.8. Peak Resolution and Column Efficiency

The influence of several experimental conditions including the type and concentration of chiral selector, the concentration of SDS, the ratio of methanol, the concentration of sodium borate, and the separation voltage was investigated. The optimal conditions were as follows: capillary column: 50 *μ*m × 80 cm, 60 cm effective length; gravity injection: 10 s; voltage: +13 kV; buffer: 30 mM sodium borate, 20 mM SDS, 10 mM HP-*γ*-CD, 8% methanol; wavelength: 220 nm; at room temperature; sample concentration: 0.1 mg/mL diniconazole, 0.05 mg/mL hexaconazole, and 0.05 mg/mL imazalil. Meanwhile, the peak efficiency (*N*) and resolution peaks were analyzed based on the chromatogram. It can be seen from Table 1 that an HP-*γ*-CD-MEKC method for the simultaneous enantioseparation of diniconazole, hexaconazole, and imazalil had good efficiency (*N* ˃ 560000) and resolutions between two adjacent peaks. The resolutions of diniconazole, hexaconazole, and imazalil were 15.2, 2.12, and 2.78, respectively, which showed that the method met the requirement of enantiomeric separation for the three kinds of chiral antifungal pesticides.

## 4. Conclusions

Based on micellar electrokinetic chromatography (MEKC) including HP-*γ*-CD as chiral selector, chiral separation for three kinds of chiral antifungal pesticides (diniconazole, hexaconazole, and imazalil) was successfully achieved for the first time; the minimum chiral resolution for a pair of enantiomers was 2.12, and the maximum resolution was 15.2. This study provides an alternative way to systematically separate chiral antifungal pesticides with simplicity and practicability, high efficiency, and good resolution, and chiral columns and coat capillaries are not needed to be prepared. It can provide help for the study of enantiomers of diniconazole, hexaconazole, and imazalil in environmental behavior, biotoxicity, and so on. However, this method also has some shortcomings because the traditional capillary electrophoresis sample injection method has some disadvantages, which leads to the poor repeatability of the experimental method. It is expected to use quantitative capillary electrophoresis to improve repeatability. Meanwhile, the experimental method will be further verified to study the actual samples.

## Figures and Tables

**Table 1:** Peak efficiencies (*N*) and resolution between two adjacent peaks.

Peak name	Diniconazole I	Imazalil I	Imazalil II	Hexaconazole I	Hexaconazole II	Diniconazole II
Efficiencies (*N*)	797380	566825	574017	630572	647442	703205
Resolution between two adjacent peaks	—	4.67	2.78	2.43	2.12	1.82

## Data Availability

The data used to support the findings of this study are available from the corresponding author upon request.

## Abbreviations

HP-*β*-CD: Hydroxypropyl-*β*-cyclodextrin
HP-*γ*-CD: Hydroxypropylated-*γ*-CD
S-*β*-CD: Sulfated-*β*-CD
SFC: Supercritical fluid chromatography
*α*-CD: *α*-cyclodextrin
*β*-CD: *β*-cyclodextrin
*γ*-CD: *γ*-cyclodextrin.
